# Iranian Society of Cardiac Surgeons COVID-19 task force version II, restarting elective surgeries

**DOI:** 10.34172/jcvtr.2020.28

**Published:** 2020-07-22

**Authors:** Alireza Alizadeh Ghavidel, Mohammadreza Mirzaaghayan, Mohammad Ali Yousefnia, Esmaeil Asdaghpour, Ramin Baghaei Tehrani, Naser Jalilifar, Hasan Radmehr, Mahmoud Shirzad, Nicholas Austine, Hosein Ahmadi, Zargham Hossein Ahmadi, Abbas Afrasiabi Rad, Ahmadali Amirghofran, Ahmad Amin, Zahra Ansari Aval, Kamran Babazadeh, Alireza Bakhshandeh, Bahador Baharestani, Rezayat Parvizi, Amirnaser Jadbabaei, Alireza Jahangirifard, Saeed Hoseini, Manouchehr Hekmat, Amanollah Heidari, Minoosh Shabani, Parham Sadeghipour, Mehrdad Salehi, Shervin Ziabakhsh Tabari, Mohammad Abbasi, Maziar Gholampour Dahaki, Ata Firouzi, Mojgan Laali, Mohammad Hosein Mandegar, Mohsen Mirmohammadsadeghi, Mohammadali Navvabi Shirazi, Akbar Nikpajooh

**Affiliations:** ^1^Heart Valve Disease Research Center, Rajaei Cardiovascular Medical & Research Center, Iran University of Medical Science, Tehran Iran; ^2^Department of Cardiac Surgery, Tehran Children Hospital, Tehran University of Medical Science, Tehran Iran; ^3^Department of Cardiac Surgery, Day General Hospital, Tehran, Iran; ^4^Department of Cardiac Surgery, Shahid Chamran Hospital, Tehran, Iran; ^5^Department of Cardiac Surgery, Shahid Moddares Hospital, Shahid Beheshti University of Medical Science, Tehran Iran; ^6^Department of Cardiac Surgery, Rasoul Akram Hospital, Iran University of Medical Science, Tehran, Iran; ^7^Department of Cardiac Surgery, Tehran Heart Center, Tehran University of Medical Science, Tehran Iran; ^8^Editoral Office, Iranian Society of Cardiac Surgeons, Tehran, Iran; ^9^Department of Cardiac Surgery, Masih Daneshvari Hospital, Shahid Beheshti University of Medical Science, Tehran Iran; ^10^Cardiovascular Research Center, Tabriz University of Medical Science, Tabriz, Iran; ^11^Department of Cardiac Surgery, Shahid Faghihi Hospital, shiraz University of Medical Science, Shiraz, Iran; ^12^Department of Cardiology, Rajaei Cardiovascular Medical & Research Center, Iran University of Medical Science, Tehran Iran; ^13^Department of Cardiac Surgery, Milad General Hospital, Tehran, Iran; ^14^Department of Cardiac Surgery, Imam Khomeini Medical Center, Tehran University of Medical Science, Tehran Iran; ^15^Department of Cardiac Surgery, Rajaei Cardiovascular Medical & Research Center, Iran University of Medical Science, Tehran Iran; ^16^Lung Transplantation Research Center, National Research Institute of Tuberculosis and Lung Diseases (NRITLD), Shahid Beheshti University of Medical Sciences, Tehran, Iran; ^17^Department of Cardiac Surgery, Golestan Hospital, Ahvaz University of Medical Science, Ahvaz Iran; ^18^Department of Infectious Disease, Loghman Hospital, Shahid Beheshti University of Medical Sciences, Tehran, Iran; ^19^Cardiovascular Intervention Research Center, Rajaie Cardiovascular, Medical, and Research Center, Iran University of Medical Sciences, Tehran, Iran; ^20^Department of Cardiac Surgery, Fatemeh Zahra Hospital, Mazandaran University of Medical Science, Sari Iran; ^21^Department of Cardiac Surgery, Imam Reza Hospital, Mashhad University of Medical Science, Mashhad Iran; ^22^Department of Cardiac Surgery, La pitié-salpetrier Hospital, Paris, France; ^23^Department of Cardiac Surgery, Shariati hospital, Tehran University of Medical Science, Tehran Iran; ^24^Department of Cardiac Surgery, Shahid Chamran Hospital, Isfahan University of Medical Science, Isfahan Iran; ^25^Department of Cardiac Surgery, Laleh Hospital, Tehran Iran; ^26^Department of Social Medicine, Rajaei Cardiovascular Medical & Research Center, Iran University of Medical Science, Tehran Iran

**Keywords:** COVID-19, Corona, SARS-CoV-2, Cardiac Surgery, COVID-19 PPE, Task Force

## Abstract

Given the nature of heart disease and the importance of continuing heart surgery during the pandemic and its aftermath and in order to provide adequate safety for the surgical team and achieve the desired result for patients, as well as the optimal use of ICU beds, the medical team, blood, blood products, and personal protective equipment, it is essential to change the usual approach during the pandemic. There are still a lot of evidences and experiences needed to produce the perfect protocol. Some centers may have a special program for their centers during this period of epidemics that can be respected and performed. Generally, in pandemic conditions, the use of non-surgical approaches is preferred if similar outcomes can be obtained.

## Introduction

In COVID-19 pandemic, the first peak is declining in many countries, and gradual return to normal social activity is the governmental plan in many areas. Adequate safety for the surgical team and obtaining optimal result for patients are two main key points in cardiac surgery. According to the situation during the first weeks of COVID-19 hit, the Iranian Society of Cardiac Surgeons have put together a task force to classify patients and provide a protocol for cardiac surgeons to perform safe surgeries both for the medical staff and the patients during the pandemic. The main focus of the first version of the protocol was on urgent and emergent cases and the basic personal protection principles; however as the pandemic is slopping down, it is time to start elective surgeries with proper precautions again.^1^ We hope that by using the new approach, while continuing to provide the best quality of care for heart patients, the existing resources will be used in the best way and the health of the medical team will be properly guaranteed. Although medical knowledge and information about this pandemic disease is becoming more complete every week, unfortunately the share of our country’s science production is low and to prepare the protocol, we must be content with other countries’ articles, which in some cases may not be sufficiently consistent with the opinion and experience of our colleagues in this specific situation of Iran. There are still a lot of evidences and experiences needed to produce the perfect protocol. Some centers may have a special program for themselves during this period of epidemics that can be respected and performed, however we hope that our knowledgeable and hardworking colleagues publish their recent studies and scientific experiences and help us to provide a more desirable national protocol.

Generally, in pandemic conditions, in case of obtaining similar clinical results, it is better to use non-surgical strategies when feasible.^2^ To facilitate clinical judgment, patients are divided into three classifications: emergent, urgent, and elective.^3^

1. **Emergent surgeries:** Life-saving cardiac surgery candidates such as patients with cardiac stab wound, mechanical valve thrombosis, type A acute aortic dissection and coronary artery disease (CAD) patients with ongoing ischemia not responsive to optimal medical or interventional approaches, that needs to be performed within hours after diagnosis (Table S1 in Supplementary file 1)
2. **Urgent surgeries:** Surgeries that should be performed within days or in the same hospital setting after diagnosis such as patients who need cardiac surgery due to heart failure secondary to the structural heart disease who are temporarily controlled with medical treatment, or CAD patients who had low threshold angina despite optimal medical treatment (Table S1 in Supplementary file 1).
3. **Elective surgeries:** Non-life-saving surgeries that can be postponed according to the heart team decision such as chronic mitral regurgitation, atrial septal defect and stable and controlled CAD by non-surgical methods.

## II. Principles of Personal Protection^4,5^

1. If there is a steady decrease in the incidence of new cases of COVID-19 in that area (province and city) for 14 consecutive days
2. Enough number of ventilator, personal protective equipment (PPE), ICU beds, blood and blood products available for the cardiac surgery team.
3. A ready and well-trained heart surgery team to be available in terms of the principles of using PPE.
4. It is recommended that elective surgeries re-start with partial capacity of operating rooms and ICUs, and the possibility of the second peak of the disease should also be considered. Also consider allocating specialized operating rooms and ICUs for patients with COVID-19.
5. Laboratory tests and radiographic images should be performed based on the indications and conditions of each patient. Routine use and repeating the above diagnostic methods without indication is not recommended.
6. Whenever possible, allocate an operating room with optimal conditions, considering the infrastructure and potential facilities of each center and pay special attention to the application of negative pressure, proper ventilation, proper discharge of exhaust gases from heart and lung pumping devices, anesthesia machine and ventilators, use of central suction and optimal observance of social distance in operating rooms.

## II. Principles of Personal Protection

The general instructions for personal protection have not changed much since the beginning of the pandemic and it is generally recommended to keep treating patients with proper precautions regardless of the slope of the epidemic.^1^

## III. Cardiac Complications of COVID-19 Patients Who Need Cardiac Surgery

1. Massive pericardial effusion/tamponade: Pericardial drainage is recommended for patients whose fluid cannot be drained by percutaneous drainage. In order to reduce the possibility of contamination of the surgical team, surgery under general anesthesia seems to be a more appropriate choice. subxiphoid drainage is not preferred without general anesthesia.
2. Acute and severe heart failure secondary to multiple organ failure placement of intra-aortic balloon pump (IABP) and extracorporeal membrane oxygenation (ECMO) can be used for these patients based on clinical conditions and heart team decisions. Peripheral and preferably percutaneous arteriovenous ECMO in conjunction with IABP can be used as a destination or bridge adjunctive.
3. Cardiomyopathy secondary to COVID-19 involvement that does not respond appropriately to medical and invasive optimum therapies. Cardiomyopathy secondary to COVID-19 is often the result of multiple organ failure, however if cardiomyopathy occurs secondary to COVID-19 involvement independently and the patient is hemodynamically unstable despite the guideline directed medical therapy (GDMT) as well as invasive procedures such as balloon pump and ECMO (INTERMACS I, II), Cardiac transplantation may be considered. Decision making in these patients is based on the prognosis of the COVID-19 disease, the patient’s life expectancy, the facilities and experiences of each center and is made with the decision of the heart and COVID team together (including the heart team, infectious disease specialist, pulmonologist, heart anesthesiologist and intensive care specialist).
4. Role of ECMO in COVID-19 patients: Not many studies have been published in this regard, although ARDS from previous SARS epidemics has reported success in using ECMO, as well as reports of the usefulness of ECMO in the treatment of severe cases of COVID-19 disease.^6^ However, the results of different countries do not match well, and the major benefits of ECMO have been reported from China and Europe, especially France. There is no published study from our country, but the experiences of the colleagues of Masih Daneshvari Hospital are valuable, however the results do not seem satisfactory.^7^

At present, it is not possible to determine specific criteria for using ECMO with the available national information, but it is clear that ECMO should be used in special patients, based on heart team decision, by the trained team, in a center with sufficient ECMO experience for non-COVID patients. Its use is not recommended in centers without sufficient experience and background.

## IV. Cardiac Surgery in COVID-19 Patients

1. Emergent/urgent surgeries: It is clear that all Emergent and Urgent procedures must be performed with safety precautions during this outbreak. In patients with COVID-19 who need Emergent or Urgent heart surgery, the COVID-19 team will make the decision based on patients’ current clinical status and comorbidities.^1^ Since the morbidity and mortality of such patients are significantly high, in order to optimize the surgical results and minimize the risk of infectivity for the medical staff, it is suggested to postpone all urgent COVID-19 candidates whenever logical based on the clinical status of the patient until acceptable recovery of the patient from the viral infection.^4,5,8^
2. Elective surgeries: Elective surgeries of waiting list patients who are involved with COVID-19 infection should be postponed until clinical and laboratory recovery from COVID-19 involvement is ensured. Obviously, if the patient’s clinical condition changed and indicates an emergent or urgent operation, principles of paragraphs I-4 should be followed. Elective surgeries in patients with COVID-19 could be performed safely whenever they have two negative PCR tests (from nasopharyngeal secretions) at least 24 hours apart, have no fever without the use of antipyretic and also have no respiratory symptoms for at least 72 hours. In addition, for elective surgery in patients with a history of COVID-19 infection, the functional status and respiratory capacity of patients should also be assessed as standard preoperative evaluations. In asymptomatic patients with positive COVID-19 PCR test, surgery should be postponed for at least 14 days. After this period, the PCR test should be done two times for at least 24 hours apart before final decision making. Because about 40% of people who test positive for COVID-19 are asymptomatic, screening for asymptomatic people who are candidates for elective surgery is also recommended. It should be noted that the sensitivity of the COVID test (PCR) is reported to be 70%-90%, and there is a 30% false negative, which is due to the time of involvement with the virus, type of kit, sampling challenges especially in old aged patients due to cooperation difficulties for proper sampling. Transmission of the virus is also possible three days before the onset of the disease. Virus accumulation is usually no longer detectable 21 days after the onset of symptoms; however, the patient’s test may remain positive in severe infections, even after this time.
Performing ELISA tests for serum antibody levels (IgM and IgG) is not recommended as a routine screening method because of cross-interaction with other Corona viruses. Additionally these antibodies can be usually measured in the second week after the onset of symptoms but not in all patients. However, due to the low cost and availability of this method and the increasing numbers of asymptomatic COVID-19 virus carriers, including medical staff, the proper and case by case use of this method in the clinical practice can be valuable.
3. Principles of personal protection in cardiac surgery of patients with COVID-19^9,10^: The recommended personal protection for COVID patients include the use of full body protection with goggles, respirator or a surgical mask on top of an N95 mask and two surgical gloves with strict use of PPE. Preferably use an operating room with negative pressure allocated for COVID-19 patients and using the central surgical suction for evacuation of electrocautery emitted gases. It is recommended to limit the presence of personnel in the operating room, specifically while inducing anesthesia and cardiopulmonary resuscitation process.^1^ Before the operation, make sure that the safe system for evacuation of anesthetic gases, pipes and disposable items are working properly.

## V. Healthy Looking People (From COVID-19 view) Candidate for Cardiac Surgery During Pandemic

As a general recommendation these patients should not undergo elective surgeries in referral centers for COVID-19 patients as much as possible. These patients should preferably be referred to non-referral and less involved hospitals with COVID-19 or their elective surgery should be postponed as another option. In COVID-19 referral centers, it is not forbidden to start the activity if there is a dedicated ICU for cardiac surgery and it is possible to completely isolate the cardiac surgery service from the services of COVID units.

These patients are classified into two groups of high and low risk:

### High Risk Group:

- Clinical or paraclinical (non-specific) suspicious COVID-19 patients
- Suspicious COVID-19 patients who are waiting for final PCR diagnosis
- Those who have had an unprotected social contact with a confirmed COVID-19 case
- Patients who need emergent cardiac surgery and have no time for screening tests.

### Low Risk Group:

- Patients who have no diagnostic findings in favor of COVID-19
- Lack of suspected clinical symptoms in favor of the 19-Covid patient during the last two weeks, including fever, chill, cough, malaise and muscle pain, sore throat, and new unexplained loss of smell and taste.
- Lack of recent paraclinical findings in favor of COVID-19 (Specific COVID-19 tests) or non-specific tests such as CBC, CRP, LDH.

1. Emergent Operations in High Risk Group: These procedures should be performed in all cardiac surgery centers, considering all protective principles recommended in paragraph IV-3.
2. Urgent Operations in High Risk Group: These operations can be performed in all centers in accordance with the recommended protection principles in paragraph IV-3. It is recommended to postpone the patient’s surgery for a short time - when possible and if the patient is stable- and evaluate COVID-19 involvement to determine the prognosis of cardiac surgery. Biomarkers such as troponin interleukin-6, D-dimer, and Pro BNP can also be used. These will also be helpful in informing the patient and the family. Chest CT scans should be performed in cases where it is indicated or helpful for surgical plan, recommended in preoperative consultations or based on each center’s dedicated protocol. Routine chest CT scans are not recommended as a screening method for COVID-19 virus according to the existing evidences. Measuring O2 Saturation is a simple, low-cost, and available method that can help.
3. Elective Surgeries in High Risk Group: These surgeries must be postponed for at least 14 days. After this period and complete clinical improvement, it is recommended to perform two consecutive PCR tests in duration of at least 24 hours.^12^ Then proceed according to paragraph II-4.^4,5,13^
4. Urgent and Emergent Surgeries in the Low Risk Group: These surgeries should be performed according to the surgical standards in non-epidemic period. It is recommended, if possible without delay in the surgical process, one PCR sampling be performed preoperatively and proper postoperative management be made accordingly.
5. Elective Surgeries in Low Risk Group: Different recommendations and methods can be found in different centers and studies, and there is no standard and definite method. The general recommendation is waiting for enough control of the epidemic. If possible, heart surgery of these patients should not performed in hospitals dedicated to COVID-19 patients or those seriously involved with COVID-19 patients, and be referred to medical centers in the same city where they are less likely to be involved with the virus. It is obvious that in the referral centers of COVID-19 and also in cities where there is only one cardiac surgery center, if there is a dedicated ICU for heart surgery and there is a complete isolation of the heart surgery ward from the services of COVID-19 wards, it is possible to start elective surgeries. These patients should not have suspicious contact with a COVID-19 patient (unprotected) or fever and other known clinical symptoms for two weeks prior to surgery. It is wise to start with limited number of elective surgeries until the adequate control of the epidemic. It is important to note that diagnostic and treatment facilities, the conditions of each city, each center, and the strategy of Covid Team play an important role in the preoperative evaluation method. Therefore, the following methods can be used to evaluate patients before surgery:

- Laboratory tests, including CBC diff, CRP, and LDH, along with two PCR COVID-19 tests, should be performed at least 24 hours apart. The interval between two tests should be within the incubation period of the disease and the patient’s tests should be performed in five days before the surgery.^12^
- Laboratory tests including CBC diff, CRP, LDH, along with a PCR and chest CT scan, in cases where there are other indications for it, such as assessing the condition of a patient with a history of pulmonary disease. CT scans are not recommended for children as a routine COVID-19 screening method.^14-18^ According to available sources, chest CT scans have been discouraged as a routine screening method and there is currently no reliable study from Iran, but based on the experience of colleagues and in individualized cases, chest CT scans can be performed in adults. Obviously, a simple chest x-ray routine, as a well-known preoperative procedure, can be helpful. Measuring O2 Saturation is a simple, low-cost, and available method that can be helpful.

In the high-risk group, if it is not possible to delay surgery due to clinical conditions, it is recommended that surgery be performed in the operating room dedicated for COVID-19 patients if possible, and then the patient be transferred to the ICU-OH assigned to COVID patients.^19^

Patients in the low-risk group can be operated under standard conditions in cardiac surgery, after which the patient will be cared for at the standard ICU of open heart surgeries. Due to the relatively long and variable incubation period of the disease and asymptomatic vectors and carriers, it is recommended that even in the surgery of low-risk patients in epidemic conditions, the principles of personal protection be observed more carefully and obsessively, such as full-time use of masks when attending hospital and operating room.

## VI. Summary of Preoperative Evaluation before Elective Cardiac Surgery

1. All patients should be carefully evaluated for clinical symptoms as well as unprotected contact in the last two weeks, and symptomatic patients should be referred to the COVID team.
2. Two PCR COVID tests should be performed at least 24 hours apart for adults and at least once for children.
3. Due to the existence of false negative cases of COVID test (about 30%) and asymptomatic patients, it is recommended that the surgical team and operating room staff always use a surgical mask and goggles, and to perform the actions that produce aerosol, use N95 mask, gown and gloves.
4. Elective surgery for patients who have a positive COVID test should be postponed until the disease is completely cured.
5. COVID-19 positive patients are considered free of infection and contamination when:
- Have two negative COVID-19 tests with an interval of at least 24 hours.
- Do not have a fever for at least 72 hours without using antipyretic drugs.
- Respiratory symptoms are completely resolved.
6. For elective surgery in patients with a history of COVID-19 disease, in addition to paragraphs IV and V, the functional and respiratory capacity of patients should be evaluated and considered for the decision to perform elective surgery.
7. Pulmonary CT scan is not recommended as a routine screening method for COVID-19 involvement.
8. Observing the social distance in the whole course of treatment between the patient and the family, other patients and the treatment team plays an important role in the health of patients.

**Figure 1:**
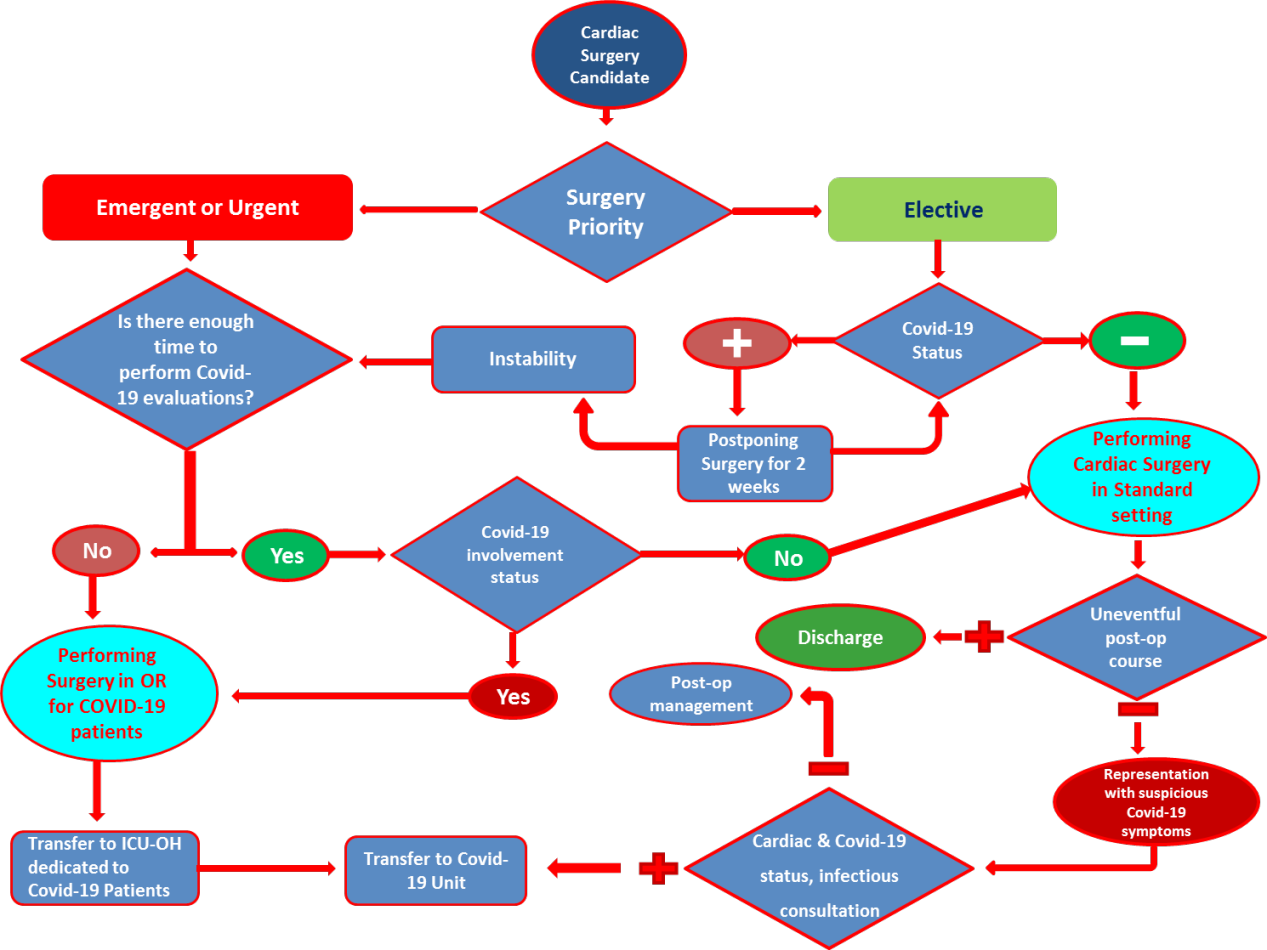
Algorithm for the management of cardiac surgery patients during COVID-19 outbreak

The presented algorithm summarizes the diagnosis and treatment process (Figure 1).

## VII. Informing Consensus for Cardiac Surgeries During Pandemic

Given the probable risks of cardiac surgeries and the possibility of COVID-19 involvement after the surgery due to false negative results during the pandemic, there are certain limitations that should be fully explained to the patients’ family and informed written consent needs to be obtained.^1^

## VIII. Blood and Blood Products Transfusion During Pandemic

The evidences are not sufficient and no infections have been reported to date confirming COVID-19 infection through being in contact with a person’s blood or transfusion of blood or blood products. However the overall recommendation is to respect all patient blood management strategies including performing a meticulous surgery to limit the need for more transfusion.^1,21^

## IX. Principles of Cardiopulmonary Resuscitation in Patients After Heart Surgery

The recommended protocol has been prepared by the Iranian Heart Association which can be referred to.^22^

## X. Heart failure surgery (Heart Transplant and Ventricular Assist Device Implantation)

Since heart transplant candidates are more prone to the COVID-19 complications, it is recommended to limit heart failure surgeries to only critically ill patients (Intermacs I and II) as lifesaving procedures.^1^

## XI. How to Deal with Patients Who Have Suspicious Symptoms of COVID-19 Involvement After Cardiac Surgery

Since post-operative complications of cardiac surgery may mimic the symptoms of COVID-19, therefore in case of presentation of similar clinical symptoms during the recovery period, it is recommended to firstly rule out cardiac complications by hospitalizing the patient in an isolated bed for suspicious cases and then check for COVID-19 involvement status.^1^ Appropriate approach must be performed based on final diagnosis.

## XII. Patient Follow-up After Discharge

In order to minimize contact and keep both patients and medical staff safe and maintain enough post-operative care, it is recommended to perform the follow-ups by telemedicine or by telephone as much as possible.

## Conclusion

Amid coronavirus pandemic, as the epidemic emerged in the world, limited knowledge and resources and the need for providing medical services for cardiovascular patients in various stages has lead us to conduct ways to provide adequate service while protecting patients, medical staff and our resources like blood atynd blood products. As there are no standard source of knowledge to be referred to for medical centers, each hospital has put together a protocol to keep servicing cardiovascular patients. The general recommendation is to delay or choose non-surgical approaches if possible. Obviously, emergent and lifesaving operations like acute type A aortic dissection should be performed with full personal protection and written informed consent should be obtained from patients’ family. Urgent surgeries are suggested to be delayed if possible, otherwise pre-operative diagnostic measures including blood and Covid-19 PCR samples should be taken and after the surgery proper decisions will be made according to the test results. To provide surgical services to elective patients, they were divided into two categories of low risk and high risk groups based on Covid-19 involvement. Patients who are suspicious for Covid-19 infection, have had unprotected contact with Covid-19 positive cases or are waiting for Covid-19 PCR results are considered high risk. These surgeries should be postponed for at least 14 days, patient reassessment and two consecutive negative Covid-19 PCR tests. For elective surgeries in low risk patients the general recommendation is waiting for enough control of the epidemic. If possible, heart surgery of these patients should not performed in hospitals dedicated to COVID-19 patients or those seriously involved with COVID-19 patients, and be referred to medical centers in the same city where they are less likely to be involved with the virus. To sum up, there are risks for both patients and medical staff during the pandemic and therefore medical staff should consider protective measures according to protocols regardless, and the families of patients need to receive enough information.

## Competing interests

None to declared.

## Ethical approval

Not applicable.

## Funding

None.

## Supplementary materials


Supplementary file 1 contains Table S1.

## References

[1] Asdaghpour E, Baghaei R, Jalilifar N, Radmehr H, Shirzad M, Alizadeh Ghavidel A (2020). Iranian Society of Cardiac Surgeons Position Statement for the Treatment of Patients in Need of Cardiac Surgery in the COVID-19 Pandemic Period (Version I). Multidiscip Cardio Annal.

[2] Iranian Health Ministry. National. Flowchart of dignosis and treatment of COVID-19 in outpatient and inpatient settings 2020 [updated March 25th 2020]. Available from: https://irimc.org/Portals/0/NewsAttachment.

[3] Stahel PF (2020). How to risk-stratify elective surgery during the COVID-19 pandemic?. Patient Saf Surg.

[4] The ASA and APSF Joint Statement on Perioperative Testing for the COVID-19 Virus. Available from: https://www.asahq.org/about-asa/newsroom/news-releases/2020/04/asa-and-apsf-joint-statement-on-perioperative-testing-for-the-covid-19-virus.

[5] Joint Statement: Roadmap for Resuming Elective Surgery after COVID-19 Pandemic. Available from: https://www.asahq.org/about-asa/newsroom/news-releases/2020/04/joint-statement-on-elective-surgery-after-covid-19-pandemic.

[6] Ryan C (2019). Maves, Christina M Jamros, Intensive Care Unit Preparedness during Pandemics and Other Biological Threats. Crit Care Clin.

[7] Ramanathan K, Antognini D, Combes A, Matthew Paden, Bishoy Zakhary, Mark Ogino (2020). Planning and provision of ECMO services for severe ARDS during the COVID-19 pandemic and other outbreaks of emerging infectious diseases. Lancet Respir Med.

[8] Lei S, Jiang F, Su W, Chen C, Chen J, Mei W (2020). Clinical characteristics and outcomes of patients undergoing surgeries during the incubation period of COVID-19 infection Version 2. EClinicalMedicine.

[9] Ti LK, Ang LS, Foong TW, Ng BSW (2020). What we do when a COVID-19 patient needs an operation: operating room preparation and guidance. Can J Anaesth.

[10] Hassan A, Arora RC, Adams C, Bouchard D, Cook R, Gunning D (2020 Jun). Cardiac Surgery in Canada During the COVID-19 Pandemic: A Guidance Statement From the Canadian Society of Cardiac Surgeons. Can J Cardiol.

[11] He Y, Wei J, Bian J, Guo K, Lu J, Mei W (2020). Chinese Society of Anesthesiology Expert Consensus on Anesthetic Management of Cardiac Surgical Patients With Suspected or Confirmed Coronavirus Disease 2019. J Cardiothorac Vasc Anesth.

[12] ASA and APSF Joint Statement on Perioperative Testing for the COVID-19 Virus. Available from: https://www.asahq.org/about-asa/newsroom/news-releases/2020/04/asa-and-apsf-joint-statement-on-perioperative-testing-for-the-covid-19-virus.

[13] Al-Muharraqi MA (2020). Testing recommendation for COVID-19 (SARS-CoV-2) in patients planned for surgery - continuing the service and ‘suppressing’ the pandemic. Br J Oral Maxillofac Surg.

[14] Liu Z, Zhang Y, Wang X, Zhang D, Diao D, Chandramohan K (2020). Recommendations for surgery during the novel coronavirus (COVID-19) epidemic. Indian J Surg.

[15] Brücher BL, Nigri G, Tinelli A, Jose Florencio F (2020). Lapeña JFF jr, Espin-Basany E, et al COVID-19: Pandemic surgery guidance. 4open.

[16] Revel MP, Parkar AP, Prosch H, Silva M, Sverzellati N, Gleeson F (2020). COVID-19 patients and the radiology department - advice from the European Society of Radiology (ESR) and the European Society of Thoracic Imaging (ESTI). Eur Radiol.

[17] Yang W, Sirajuddin A, Zhang X, Liu G, Teng Z, Zhao S (2020). The role of imaging in 2019 novel coronavirus pneumonia (COVID-19). Eur Radiol.

[18] Meng H, Xiong R, He R, Lin W, Hao B, Zhang L (2020). CT imaging and clinical course of asymptomatic cases with COVID-19 pneumonia at admission in Wuhan, China. J Infect.

[19] Ti LK, Ang LS, Foong TW, Ng BSW (2020). What we do when a COVID-19 patient needs an operation: operating room preparation and guidance. Can J Anaesth.

[20] Aminian A, Safari S, Razeghian-Jahromi A, Ghorbani M, Delaney CP (2020 Jul). COVID-19 Outbreak and Surgical Practice: Unexpected Fatality in Perioperative Period. Ann Surg.

[21] Kwon SY, Kim EJ, Jung YS, Jang JS, Cho NS (2020). Post-donation COVID-19 identification in blood donors. Vox Sang.

[22] Barati S, Garjani K, Payandemehr P, Totonchi Z, Zanganehfar ME, Sadeghipour P (2020). Iranian heart association task force on cardiopulmonary resuscitation guidelines on the COVID-19 outbreak. Res Cardiovasc Med.

