# Supplementary material for: Iranian Society of Cardiac Surgeons COVID-19 task force version II, restarting elective surgeries
Source: J Cardiovasc Thorac Res. 2020 Jul 22;12(3):158–64. doi: 10.34172/jcvtr.2020.28 (PMC7581843; doi:10.34172/jcvtr.2020.28)
Supplement: Supplementary file 1 — contains Table S1. [file jcvtr-12-158-s001.pdf]

Supplementary file 1

Table S1. Cardiac operations classification based on level of urgency during COVID 19 pandemic in Iran

Emergent Operations	Urgent Operations
Cardiac & Great artery Trauma	CAD patient with low threshold angina (Class III-IV)
Acute Type A Dissection	Patients with severe symptomatic AS
Cardiac Tamponade	NVE or PVE after optimal Antibiotic and medical therapy or complicated endocarditis
Mechanical Valve Thrombosis	Acute MR (Flail MV)
Acute Pulmonary Emboli<br>(with no medical or interventional options)	Post-MI complications (VSD,LV false aneurysm, sever acute MR)
Patients with ACS and sever CAD<br>(Significant LM lesions or high SYNTAX score)	Post-operative Mediastinitis
Post PCI or other percutaneous approaches sever complications	CAD patients with ostial LAD lesion amenable to percutaneous management
Ongoing ischemia in CAD Patients uncontrolled by percutaneous approaches and GDMT	Large right heart clots (>20mm) with no other non-surgical treatment option
Patients with STEMI not suitable response to GDMT or percutaneous approaches (or have ongoing ischemia after culprit lesion PCI)	Large hypermobile left heart clots (>10mm) with no other non-surgical treatment option
CHD patients with cyanotic spell	LA Myxoma
Prostaglandin dependent CHD patients	Ascending Aorta aneurysm larger than 60 mm
Individualized cases (according to the heart team decision making)	Subacute and Chronic Type A dissection
